# Beam Allocation and Power Optimization for Energy-Efficiency in Multiuser mmWave Massive MIMO System

**DOI:** 10.3390/s21072550

**Published:** 2021-04-06

**Authors:** Saidiwaerdi Maimaiti, Gang Chuai, Weidong Gao, Jinxi Zhang

**Affiliations:** School of Information and Communication Engineering, Beijing University of Posts and Telecommunications, Beijing 100000, China; chuai@bupt.edu.cn (G.C.); gaoweidong@bupt.edu.cn (W.G.); jinxi@bupt.edu.cn (J.Z.)

**Keywords:** massive MIMO, energy efficient, beam allocation, power optimization, mmWave, convex optimization scheme, lagrange dual method

## Abstract

This paper studies beam allocation and power optimization scheme to decrease the hardware cost and downlink power consumption of a multiuser millimeter wave (mmWave) massive multiple-input multiple-output (MIMO) system. Our target is to improve energy efficiency (EE) and decrease power consumption without obvious system performance loss. To this end, we propose a beam allocation and power optimization scheme. First, the problem of beam allocation and power optimization is formulated as a multivariate mixed-integer non-linear programming problem. Second, due to the non-convexity of this problem, we decompose it into two sub-problems which are beam allocation and power optimization. Finally, the beam allocation problem is solved by using a convex optimization technique. We solve the power optimization problem in two steps. First, the non-convex problem is converted into a convex problem by using a quadratic transformation scheme. The second step implements Lagrange dual and sub-gradient methods to solve the optimization problem. Performance analysis and simulation results show that the proposed algorithm performs almost identical to the exhaustive search (ES) method, while the greedy beam allocation and suboptimal beam allocation methods are far from the ES. Furthermore, experiment results demonstrated that our proposed algorithm outperforms the compared the greedy beam allocation method and the suboptimal beam allocation scheme in terms of average service ratio.

## 1. Introduction

The new technologies of massive multiple-input multiple-output (MIMO) and millimeter wave (mmWave) [1,2] play a key role in fifth-generation (5G) wireless communication systems. The massive MIMO and mmWave technologies have great potential to satisfy the requirements of current wireless communication systems, such as having a high data rate, tolerance of transmission latency, low cost, system security, and good user experience. In addition, they are supported the transmission of big data in the internet of things (IoT) networks [3,4,5,6]. The key idea of massive MIMO technique is the use of multiple antennas in the transmitter and the receiver to increase system performance and reliability through strategies, such as beam-forming, diversity transmission, spatial multiplexing, and interference suppression, meanwhile providing services for several users sharing the same spectrum resources, and the mmWave techniqueis able to provide huge amounts of mmWave frequency bandwidth and a large numbers of narrow beams with high directive gain. These features will further to enhance system energy efficiency (EE) and reduce the power consumption of networks [7,8].

In a practical network, one of the key challenges in a mmWave massive MIMO system is hardware cost and power consumption. Since each antenna in MIMO systems usually requires a separate radio-frequency (RF) chain that consists of RF amplifiers and analog-to-digital converters, which are very pricey and huge power consumption elements in massive MIMO systems. The power consumption of RF chain becomes huge with the number of antennas growing large in a mmWave communication system [9]. Mssive MIMO technology enables 5G wireless network to be capable of generating a large number of beams. In that scenario with multiple beams, only partial beams are used to serve all users in a cell. As a result, inter-beam interference has a high dependency on beam-user association results. This makes the beam-user association problem in the mmWave massive MIMO systems much more complicated and still remains largely unknown. Additionally, in conventional beam allocation systems with a small number of beams, even when all the beams are used, most of the users are still unserved. If a user can not get service for a long time, long service delay appears and it will lead to a decline of user service quality and overall system performance, which is undesirable for the delay sensitive services [10]. Moreover, in conventional beam allocation systems, it is assumed that the power of the base station (BS) is equally allocated for each beam, but the equal power allocation strategy can not realize optimal and full use of energy resources. It is therefore of great importance for us to study the beam allocation and power optimization problem in massive MIMO systems.

MmWave communication brings higher communication bandwidth, but with large path loss. In order to compensate for the high isotropic path loss caused by high frequencies, beam-forming technology can be employed [11], as it can extend the communication range using narrow beams of high gain. When multiple narrow beam-forming vectors are configured both in a base station (BS) and user, the beam allocation process is necessary to find the best beam-pair among all possible beam pairs for maximum beam-forming efficiency.

Recently, a range of beam allocation and power optimization algorithms have been proposed for mmWave massive MIMO systems. They mainly focused on enhancing sum-rate, decreasing power consumption, and improving energy efficiency [12,13,14,15,16,17,18]. Reference [13] proposed a beam selection concept in mmWave massive MIMO systems, whereby the number of the RF chains can be reduced by exploiting beam selection scheme without obvious system performance loss. Reference [14] proposed two analog beam selection schemes with low complexity to enhance the EE in the hybrid massive MIMO systems. Reference [15] proposed a two-step low complexity beam allocation scheme to reduce the signaling overhead while maximizing the sum date rate. Reference [16] studied the maximizing system sum rate and decreasing downlink power consumption performance of multiuser switched-beam mmWave massive MIMO system by applying a low complexity beam allocation algorithm with a limited number of RF chains. Reference [17] proposed an adaptive frequency-reuse based beam allocation algorithm to reduce the rate disparity of the users and improve the individual data rates of the worst-case users. Reference [18] proposed a joint beam selection method in mmWave massive MIMO systems with discrete lens array using equal power allocation. In [18], the authors mainly considered the channel correlation among users to enhance system sum rate. In addition, Reference [19] proposed a joint power allocation and beam-forming algorithm to maximize the sum rate in a multiuser mmWave-NOMA system. However, all the previous works [13,14,15,16,17,18] assumed that the power of the BS is equally allocated for each beam, but the equal power allocation strategy can not realize the optimal and full use of energy resources. Therefore, studying an efficient beam allocation and power optimization algorithm for EE is important to improving system performance.

In this paper, we study the beam allocation and power optimization problems in a multiuser massive MIMO downlink system working at the mmWave frequency band. The main contributions of our work are as follows:

- In this paper, we proposed beam allocation and power optimization algorithm to enhance EE without obvious system performance loss. First, the problem of beam allocation and power optimization is formulated as a multivariate mixed-integer non-linear programming problem. Second, because of the non-convexity of this problem, we decompose it into two sub-problems, which are beam allocation and power optimization;
- The beam allocation problem is solved by a convex optimization technique. For power optimization, first, the non-convex problem is converted into a convex problem by using a quadratic transformation scheme. After that, we used Lagrange dual and sub-gradient methods to solve the convex problem;
- Our experiments demonstrate that the proposed algorithm performs almost identical to ES method, and surpasses both the greedy beam allocation method and the suboptimal beam allocation scheme in terms of EE and average service ratio.

Notation: Symbol E[·] denotes the expectation operator; vectors are denoted by using lower-case bold letters and matrices are denoted by using bold letters; and $\left|\cdot \right|$ denotes the absolute value of a scalar.

The remaining content is organized as follows. Section 2, describes the system model and formulates the energy-efficiency maximizing problem. In Section 3, we discuss the proposed algorithm based on different schemes. Section 4 provides the simulation results and analyzes the computational complexity. Finally, this work is concluded in Section 5.

## 2. System Model and Problem Formulation

### 2.1. System Model

The considered scenario is a single-cell multiuser massive MIMO downlink system working at a mmWave frequency band, composing of an *M*-antennas BS and *U* single antenna UE (M >> U). BS is located at the cell center, where the *M* antennas at the BS will formed *M* massive number beams and employs $M_{RF}$ RF chains ($M_{RF}\;\leq \;U$) to serve *U* users, where *U* users are uniformly distributed within a circular cell with a unit radius, and the location of user *u* is at the $\Upsilon_{u}=\left(\rho_{u},\;\theta_{u}\right)$. In addition, we define $c_{m,u}\;\in \;\left\{0,\;1\right\}$ as the beam-user associate indicator. If beam *m* is allocated to user *u*, $c_{m,u}\;=\;1$; otherwise, $c_{m,u}\;=\;0$. We assume that each user can only associate with one best beam for its date transmission, and each associated beam is allocated to its one best associated user, respectively. Therefore, the constraints can be written as:

(1) $$\begin{array}{cc} \; & \sum_{m=1}^{M}c_{m,u}\leq 1,\;\;c_{m,u}\in \left\{0,1\right\},\forall u\in \left\{1,2,\ldots ,U\right\} \end{array}$$

(2) $$\begin{array}{cc} \; & \sum_{u=1}^{U}c_{m,u}\leq 1,\;\;c_{m,u}\in \left\{0,1\right\},\forall m\in \left\{1,2,...,M\right\}. \end{array}$$

### 2.2. Problem Formulation

According to Shannon’s formula, the achievable downlink rate of the *u*-th user which is served by *m*-th beam can be written as:

(3) $$\begin{array}{cc} \; & R_{m,u}=\log_{2}\left(1+\mathrm{SINR}{}_{m,u}\right) \end{array}$$

where SINR${}_{m,u}$ can be written as:

(4) $$\begin{array}{cc} \; & \mathrm{SIN}R_{m,u}=\frac{p_{m}D_{m}\left(\theta_{u}\right)\rho_{u}^{-\alpha }}{\sum_{i=1,i\neq m}^{M}p_{i}D_{i}\left(\theta_{u}\right)\rho_{u}^{-\alpha }+\sigma_{0}^{2}} \end{array}$$

where $D_{m}\left(\theta \right)$ is given by [17]:

(5) $$\begin{array}{cc} \; & D_{m}\left(\theta \right)=\frac{\sin^{2}\left(0.5M\pi cos\theta -\beta_{m}\right)}{M\sin^{2}\left(0.5\pi cos\theta -\frac{1}{M}\beta_{m}\right)} \end{array}$$

where:

(6) $$\begin{array}{cc} \; & \beta_{m}=\left(-\frac{M+1}{2}+m\right)\pi \end{array}$$

where $p_{m}\;\in$ p is the transmit power allocated to active beam *m*, where p = $\left\{p_{1},p_{2},\ldots ,p_{M}\right\}$ is the set of transmit powers. The $D_{m}\left(\theta_{u}\right)$ denotes the directivity gain of *m*-th beam in the cell at $\theta_{u}$, where $\theta_{u}$ denotes the angle of departure (AoD) of the transmitted signal for user *u*. $\rho_{u}$ denotes the distance from user *u* to the base station and α is the path-loss exponent, assuming a pure line of sight (LOS) channel at mmWave frequencies because the NLOS paths have a high propagation loss, scattering, and blockage in mmWave environments. $\beta_{m}$ denotes the array weights, we use the $\beta_{m}$ to symmetric the angular coverage of each beam with its main direction [17], and $\sigma_{0}^{2}$ denotes additive white Gaussian noise power.

Our target is to enhance system EE without performance loss. Therefore, the system EE optimization problem can be expressed as:

(7) $$\begin{array}{cc} \; & \max_{p,Q}\eta =\frac{\sum_{m=1}^{M}\sum_{u=1}^{U}c_{m,u}R_{m,u}}{\sum_{m=1}^{M}p_{m}+M\gamma } \end{array}$$

subject to:

(8) $$\begin{array}{cc} \; & \begin{array}{c} s.t.1:\sum_{m=1}^{M}c_{m,u}\leq 1,\;c_{m,u}\in \left\{0,1\right\},\forall u\in \left\{1,2,\ldots ,U\right\}\\ s.t.2:\sum_{u=1}^{U}c_{m,u}\leq 1,\;c_{m,u}\in \left\{0,1\right\},\forall m\in \left\{1,2,\ldots ,M\right\}\\ s.t.3:c_{m,u}R_{m,u}\geq R_{m,u}^{\min},\;\;\;\sum_{u=1}^{U}c_{m,u}=1\\ s.t.4:\sum_{m=1}^{M}p_{m}\leq P_{t}\\ s.t.5:\;c_{m,u}\in \left\{0,1\right\},\;\forall u\in \left\{1,\ldots ,U\right\},\forall m\in \left\{1,\ldots ,M\right\} \end{array} \end{array}$$

where Q$={\left[c_{m,u}\right]}_{M\times U}$ denotes the beam-user association matrix; η is the system EE which is nonnegative; γ is the power consumption of per transmit antenna; minimum rate of per user is denoted by $R_{m,u}^{\min}$; and $P_{t}$ is the maximum transmit power.

## 3. Beam Allocation and Power Optimization Algorithm

Due to the influence of binary variable $c_{m,u}$, the optimization Problem (7) is a typical non-convex multivariate mixed-integer non-linear programming problem and the optimum is difficult to find. Therefore, we decompose the problem into two subproblems: (1) Beam allocation problem with fixed power and (2) power optimization problem.

### 3.1. Beam Allocation

The target of beam allocation is to ensure each user can be allocated a beam. In this part, we assume the power is fixed, the denominator of optimization problem (7) is independent of optimization variables. Therefore, under the fixed power, formula (7) becomes an ‘0–1’ integer programming problem with regard to beam-user association as:

(9) $$\begin{array}{cc} \; & \max_{Q}\sum_{m=1}^{M}\sum_{u=1}^{U}c_{m,u}R_{m,u} \end{array}$$

subject to:

(10) $$\begin{array}{cc} \; & \begin{array}{c} s.t.1:\sum_{m=1}^{M}c_{m,u}\leq 1,\;c_{m,u}\in \left\{0,1\right\},\forall u\in \left\{1,2,\ldots ,U\right\}\\ s.t.2:\sum_{u=1}^{U}c_{m,u}\leq 1,\;c_{m,u}\in \left\{0,1\right\},\forall m\in \left\{1,2,\ldots ,M\right\}\\ s.t.3:c_{m,u}R_{m,u}\geq R_{m,u}^{\min},\;\;\;\sum_{u=1}^{U}c_{m,u}=1\\ s.t.4:\;c_{m,u}\in \left\{0,1\right\},\;\forall u\in \left\{1,\ldots ,U\right\},\forall m\in \left\{1,\ldots ,M\right\}. \end{array} \end{array}$$

Problem (9) is generally complicated due to the influence of binary variable $c_{m,u}$. Hence, we adopt the fractional beam-user association relaxation, where $c_{m,u}$ can take any real value in [0,1] [20] (see Lemma 1).

The relaxed beam-user association problem function:

(11) $$\begin{array}{cc} \; & \begin{array}{c} \max_{Q}\sum_{m=1}^{M}\sum_{u=1}^{U}c_{m,u}R_{m,u}\\ \;\;\;\;\;\;\;s.t.1,\;s.t.2,\;s.t.3\;in\;\left(10\right)\;,\;0\leq c_{m,u}\leq 1. \end{array} \end{array}$$

It is observed that the optimization problem (11) is a convex problem according to Lemma 1 in [20]. Hence, we apply lagrange dual method to solve this problem. The Lagrangian function of (11) is:

(12) $$\begin{array}{cc} \; & \begin{array}{c} F\left(c_{m,u},\mu_{m,u},\lambda_{u},\upsilon_{m}\right)=\sum_{m=1}^{M}\sum_{u=1}^{U}c_{m,u}R_{m,u}\\ \;\;\;\;\;\;\;\;\;\;\;\;\;\;\;\;\;\;\;\;\;\;\;\;\;+\sum_{m=1}^{M}\sum_{u=1}^{U}\mu_{m,u}\left(c_{m,u}R_{m,u}-R_{m,u}^{\min}\right)\\ \;\;\;\;\;\;\;+\sum_{u=1}^{U}\lambda_{u}\left(1-\sum_{m=1}^{M}c_{m,u}\right)+\sum_{m=1}^{M}\upsilon_{m}\left(1-\sum_{u=1}^{U}c_{m,u}\right) \end{array} \end{array}$$

where $\mu_{m,u}\;\geq \;0$, $\lambda_{u}\;\geq \;0$ and $\upsilon_{m}\;\geq \;0$ are lagrangian multipliers and they are associated with constraints s.t.3, s.t.1, and s.t.2 in (11), respectively. Correspondingly, the lagrange dual function of (11) is formulated as:

(13) $$\begin{array}{cc} \; & \begin{array}{c} D\left(\mu_{m,u},\lambda_{u},\upsilon_{m}\right)=\max_{Q}\sum_{m=1}^{M}\sum_{u=1}^{U}F_{m,u}\left(c_{m,u},\mu_{m,u},\lambda_{u},\upsilon_{m}\right)\\ \;\;\;\;\;\;\;\;\;\;\;\;\;\;\;\;\;\;\;\;\;\;-\;\sum_{m=1}^{M}\sum_{u=1}^{U}\mu_{m,u}R_{m,u}^{\min}+\sum_{u=1}^{U}\lambda {}_{u}+\sum_{m=1}^{M}\upsilon_{m} \end{array} \end{array}$$

where $F_{m,u}\left(c_{m,u},\;\mu_{m,u},\;\lambda_{u},\;\upsilon_{m}\right)$ is the corresponding Lagrange dual function for each user, and can be written as:

(14) $$\begin{array}{cc} \; & \begin{array}{c} F_{m,u}\left(c_{m,u},\mu_{m,u},\lambda_{u},\upsilon_{m}\right)=\\ \;\;\;\;\;\;\;\;\;\;\;\;\;\;\;\;\;\;\;\;\;\;\;\;\;\left(1+\mu_{m,u}\right)c_{m,u}R_{m,u}-\left(\lambda_{u}+\upsilon_{m}\right)c_{m,u} \end{array} \end{array}$$

and the lagrange dual problem of (13) can be written as:

(15) $$\begin{array}{cc} \; & \min_{\mu_{m,u},\lambda_{u},\upsilon_{m}}D\left(\mu_{m,u},\lambda_{u},\upsilon_{m}\right),\;s.t.\;\mu_{m,u}\geq 0,\lambda_{u}\geq 0,\upsilon_{u}\geq 0. \end{array}$$

According to the KKT conditions and the partial derivative of (14) with respect to variable $c_{m,u}$, we can achieve as:

(16) $$\begin{array}{cc} \; & \frac{\partial F_{m,u}\left(c_{m,u},\mu_{m,u},\lambda_{u},\upsilon_{m}\right)}{\partial c_{m,u}}=\left(1+\mu_{m,u}\right)R_{m,u}-\lambda_{u}-\upsilon_{m}. \end{array}$$

In order to achieve the maximum of (11), the best beam-user associate factor $c_{m,u}$ is defined as:

(17) $$\begin{array}{cc} \; & c_{m,u}^{}=\left\{\begin{array}{c} 1,\;\;if\;m=m_{best}\\ 0,\;if\;m\neq m_{best} \end{array}\right. \end{array}$$

where:

(18) $$\begin{array}{cc} \; & m_{best}=\underset{\forall m}{\arg\max}\left\{\left(1+\mu_{m,u}^{\left(t\right)}\right)R_{m,u}^{}-\lambda_{u}^{\left(t\right)}-\upsilon_{m}^{\left(t\right)}\right\}. \end{array}$$

We obtain the best beam-user associate factor by relaxing the binary constraint in (11). In fact, it is an optimal solution to the original problem (9) because the optimal $c_{m,u}^{}$ is fortunately either 0 or 1, which exactly satisfies constraints in (10).

Although the best beam-user associate factor is achieved, it is related to the lagrange iteration multipliers. So we update them by a sub-gradient-based method that guarantees convergence [21], and the iterations can be written as:

(19) $$\begin{array}{cc} \; & \begin{array}{c} \nabla \mu_{m,u}^{\left(t+1\right)}={\left[\mu_{m,u}^{\left(t\right)}-\delta_{1}^{\left(t\right)}\left(c_{m,u}^{\left(t\right)}R_{m,u}^{}-R_{m,u}^{\min}\right)\right]}^{+}\\ \nabla \lambda_{u}^{\left(t+1\right)}={\left[\lambda_{u}^{\left(t\right)}-\delta_{2}^{\left(t\right)}\left(1-\sum_{m=1}^{M}c_{m,u}^{\left(t\right)}\right)\right]}^{+}\\ \nabla \upsilon_{m}^{\left(t+1\right)}={\left[\upsilon_{m}^{\left(t\right)}-\delta_{3}^{\left(t\right)}\left(1-\sum_{u=1}^{U}c_{m,u}^{\left(t\right)}\right)\right]}^{+} \end{array} \end{array}$$

where ${\left[x\right]}^{+}=\max\left\{x,0\right\}$. $\delta_{1}^{\left(t\right)}$, $\delta_{2}^{\left(t\right)}$, and $\delta_{3}^{\left(t\right)}$ are iteration step sizes that should be chosen appropriately [22], i.e., δ1(t)=0.10.1tt, δ2(t)=0.10.1tt and δ3(t)=0.10.1tt.

### 3.2. Power Optimization

Given the optimized beam-user association, the optimization problem in (7) is only dependent of power variable. Therefore, we can rewrite the optimization problem in (7) as:

(20) $$\begin{array}{cc} \; & \max_{p}\eta =\frac{\sum_{m=1}^{M}\sum_{u=1}^{U}c_{m,u}R_{m,u}}{\sum_{m=1}^{M}p_{m}+M\gamma } \end{array}$$

subject to:

(21) $$\begin{array}{cc} \; & \begin{array}{c} s.t.1:c_{m,u}R_{m,u}\geq R_{m,u}^{\min},\;\;\;\sum_{u=1}^{U}c_{m,u}=1\\ s.t.2:\sum_{m=1}^{M}p_{m}\leq P_{t} \end{array}. \end{array}$$

The optimization problem (20) is a typical non-convex and nonlinear fractional programming. So it can be transformed to parametric subtractive problem by the parametric programming, and the parametric subtractive problem can be written as:

(22) $$\begin{array}{cc} \; & \max_{p}\sum_{m=1}^{M}\sum_{u=1}^{U}c_{m,u}R_{m,u}-\eta \left(\sum_{m=1}^{M}p_{m}+M\gamma \right),s.t.1,2. \end{array}$$

In addition, the rate of downlink $R_{m,u}$ is a non-convex function. So we first convert it into a convex function by applying the quadratic transformation method [23]. The objective function $R_{m,u}$ can be expressed as:

(23) $$\begin{array}{cc} \; & {\tilde{R}}_{m,u}=\log_{2}\left(1+2\varpi_{m,u}\sqrt{p_{m}D_{m}\left(\theta_{u}\right)\rho_{u}^{-\alpha }}-\varpi_{m,u}^{2}\psi_{m,u}\right) \end{array}$$

where $\psi_{m,u}\;=\;\sum_{i=1,i\neq m}^{M}p_{i}D_{i}\left(\theta_{u}\right)\rho_{u}^{-\alpha }\;+\;\sigma_{0}^{2}$, $\varpi_{m,u}$ is the auxiliary variable which is nonnegative and we can write the optimal value of $\varpi_{m,u}$ as [23]:

(24) $$\begin{array}{cc} \; & \varpi_{m,u}^{best}=\frac{\sqrt{p_{m}D_{m}\left(\theta_{u}\right)\rho_{u}^{-\alpha }}}{\psi_{m,u}}. \end{array}$$

According to reference [23] (TH.1), we can rewrite the optimization problem in (22) as:

(25) $$\begin{array}{cc} \; & \max_{p,\varpi_{m,u}}\sum_{m=1}^{M}\sum_{u=1}^{U}c_{m,u}{\tilde{R}}_{m,u}-\eta \left(\sum_{m=1}^{M}p_{m}+\gamma M\right),s.t.1^{\prime } \end{array}$$

where $s.t.1^{\prime }:c_{m,u}{\tilde{R}}_{m,u}\;\geq \;R_{m,u}^{\min},\;\;\;\sum_{u=1}^{U}c_{m,u}\;=\;1$.

It is observed that the optimization function in (25) is a convex function with respect to $p_{m}$ and $\varpi_{m,u}$, respectively. Hence, we use the Lagrangian dual scheme to address this problem. The Lagrangian function of (25) is:

(26) $$\begin{array}{cc} \; & \begin{array}{c} F^{\prime }\left(p_{m},\varpi_{m,u},\nu_{m,u},\chi \right)=\\ \;\;\;\;\;\;\;\;\;\;\;\;\sum_{m=1}^{M}\sum_{u=1}^{U}c_{m,u}{\tilde{R}}_{m,u}-\;\eta \left(\sum_{m=1}^{M}p_{m}+\gamma M\right)+\\ \sum_{m=1}^{M}\sum_{u=1}^{U}\nu_{m,u}\left(c_{m,u}{\tilde{R}}_{m,u}-R_{m,u}^{\min}\right)+\chi \left(P_{t}-\sum_{m=1}^{M}p_{m}\right) \end{array} \end{array}$$

where $\nu_{m,u}\;\geq \;0$ and χ ≥ 0 are lagrangian multipliers and are associated with constraints in (25). Correspondingly, the lagrange dual function of (25) is formulated as:

(27) $$\begin{array}{cc} \; & D\left(\nu_{m,u},\chi \right)=\max_{p,\varpi_{m,u}}F^{\prime }\left(p_{m},\varpi_{m,u},\nu_{m,u},\chi \right) \end{array}$$

and the lagrange dual problem can be written as:

(28) $$\begin{array}{cc} \; & \min_{\nu_{m,u},\chi }D\left(\nu_{m,u},\chi \right),\;s.t.\;\nu_{m,u}\geq 0,\chi \geq 0. \end{array}$$

According to the KKT conditions, we can derive the optimal power $p_{m}^{best}$ and $\varpi_{m,u}^{best}$ by equating the partial derivative of (27) to zero with respect to $p_{m}$ and $\varpi_{m,u}$, respectively. Therefore, $\varpi_{m,u}^{best}\;=\;\frac{\sqrt{p_{m}D_{m}\left(\theta_{u}\right)\rho_{u}^{-\alpha }}}{\psi_{m,u}}$ (see formula (24)), and the optimal power $p_{m}^{best}$ can be expressed as in (29), where $A\;=\;4\ln2\varpi_{m,u}\sqrt{D_{m}\left(\theta_{u}\right)\rho_{u}^{-\alpha }}$ and $B\;=\;\frac{\left(1+\nu_{m,u}^{\left(t\right)}\right)c_{m,u}}{M(\chi^{\left(t\right)}+\eta )}$.

(29) $$\begin{array}{cc} \; & \begin{array}{c} p_{m}^{best}=\frac{A^{2}{\left(1+\varpi_{m,u}^{2}\psi_{m,u}\right)}^{2}+2A\left(1+\varpi_{m,u}^{2}\psi_{m,u}\right)\sqrt{4A\sqrt{D_{m}\left(\theta_{u}\right)\rho_{u}^{-\alpha }}\left[ln2{\left(1+\varpi_{m,u}^{2}\psi_{m,u}\right)}^{2}-2B\varpi_{m,u}\right]}}{4A\varpi_{m,u}\sqrt{D_{m}\left(\theta_{u}\right)\rho_{u}^{-\alpha }}}\\ \;\;\;\;\;\;\;\;\;\;\;\;+\frac{ln2{\left(1+\varpi_{m,u}^{2}\psi_{m,u}\right)}^{2}-2B\varpi_{m,u}}{\varpi_{m,u}} \end{array}. \end{array}$$

The values of $\nu_{m,u}$ and χ is updated by a sub-gradient-based method that guarantees convergence [21], and the iterations can be written as:

(30) $$\begin{array}{cc} \; & \begin{array}{c} \nabla \nu_{m,u}^{\left(t+1\right)}={\left[\nu_{m,u}^{\left(t\right)}-\xi_{1}^{\left(t\right)}\left(c_{m,u}^{}{\tilde{R}}_{m,u}^{}-R_{m,u}^{\min}\right)\right]}^{+}\\ \nabla \chi^{\left(t+1\right)}={\left[\chi^{\left(t\right)}-\xi_{2}^{\left(t\right)}\left(P_{t}-\sum_{m=1}^{M}p_{m}^{\left(t\right)}\right)\right]}^{+} \end{array} \end{array}$$

where ${\left[x\right]}^{+}\;=\;\max\left\{x,0\right\}$. $\xi_{1}^{\left(t\right)}$ and $\xi_{2}^{\left(t\right)}$ are iteration step sizes that should be chosen appropriately [22], i.e., ξ1(t) = 0.10.1tt and ξ2(t) = 0.10.1tt.

In Algorithm 1, the two sub-problems, beam allocation and power optimization are solved in an interleave manner until convergence. For the inner loop, we calculate *Q* with a given *p*, and calculate *p* with a fixed *Q*. For the outer loop, we calculate η and update it. Note that as long as the goal of beam allocation and power optimization in each iteration is to maximize the one objective function, Algorithm 1 is guaranteed to converge.

### 3.3. Service Ratio

It should be noted that some users might not be served due to the strategy that one beam can only serve one user. If a user can not get service for a long time, long service delay appears and leads to the decline of user service quality, which is undesirable for the delay sensitive services. Hence, service delay is of great significance to evaluate system performance for massive MIMO systems. In this paper, we also study the service ratio problem to evaluate the service delay. The service delay is defined as the ratio of the number of users served to the total number of users, and can be written as:

(31) $$\begin{array}{cc} \; & S=\frac{\sum_{u=1}^{U}\sum_{m=1}^{M}c_{m,u}}{U}. \end{array}$$

In order to accurately calculate the service ratio, we further define the average service ratio because the result of beam allocation is closely related to the positions of users, and can be written as:

(32) $$\begin{array}{cc} \; & \bar{S}\overset{\Delta }{\;=}E_{\left\{\left.\Upsilon_{u}\right|u=1,2,\ldots ,U\right\}}\left[S\right] \end{array}$$

where $\Upsilon_{u}$ denotes the position of user *u*, and the average service ratio for over all possible positions of the users is denoted by $E_{\left\{\left.\Upsilon_{u}\right|u=1,2,\ldots ,U\right\}}$. Obviously, the average delay performance depends on the average service ratio. Therefore, the higher average service ratio leads to a better average delay performance.
**Algorithm 1** Proposed algorithm 1:Initialize η=0, maximum tolerance ε, *t* = 0. 2:repeat 3: Initialize any feasible p; 4: repeat
 5:  Solve beam allocation problem (9) with fixed p; 6:  Solve power optimization problem (20) with fixed Q; 7: until convergence or $t=T_{\max}$; 8: Calculate $\begin{array}{c} \varphi =\sum_{m=1}^{M}\sum_{u=1}^{U}c_{m,u}R_{m,u}-\eta \left(\sum_{m=1}^{M}p_{m}+M\gamma \right) \end{array}$ 9: Update10:$\;\;\;\;\;\eta =\left(\sum_{m=1}^{M}\sum_{u=1}^{U}c_{m,u}R_{m,u}\right)/\left(\sum_{m=1}^{M}p_{m}+\gamma M\right)$11:untilφ<ε;12:Output optimal $Q^{\mathrm{best}}$ and $p^{\mathrm{best}}$;

## 4. Simulation Results and Analsis

In this section, we evaluate the proposed algorithm and provide numerical results and behavior simulation. The key simulation parameters are summarized in Table 1.

### 4.1. Performance Evaluation

**Figure 1:**
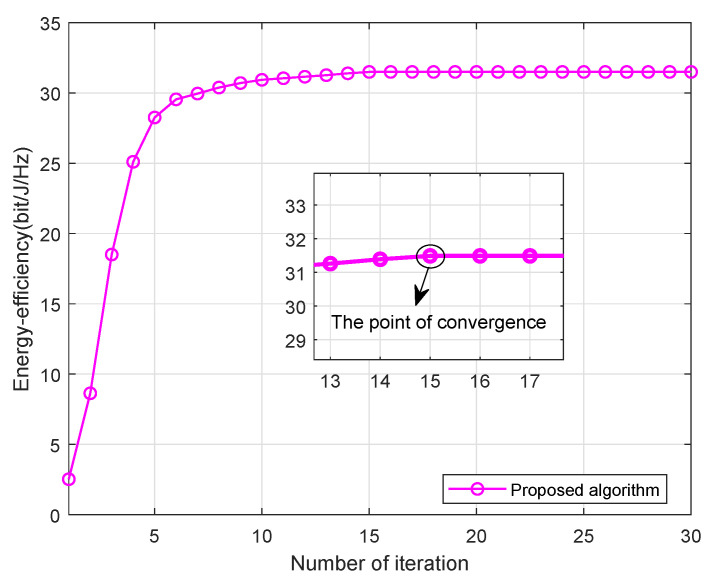
EE (energy efficiency) versus iterative with $P_{t}$ = 30 dBm, α=2, *U* = 20.

Figure 1 shows the increase of system EE during each iteration, and demonstrates the easy convergence of our proposed algorithm with $P_{t}$ = 30 dBm. As can be seen from Figure 1, the proposed algorithm converges after about t=15 iterations. As expected, the system EE obtained by our proposed algorithm monotonically increased with the number of iteration increases.

**Figure 2:**
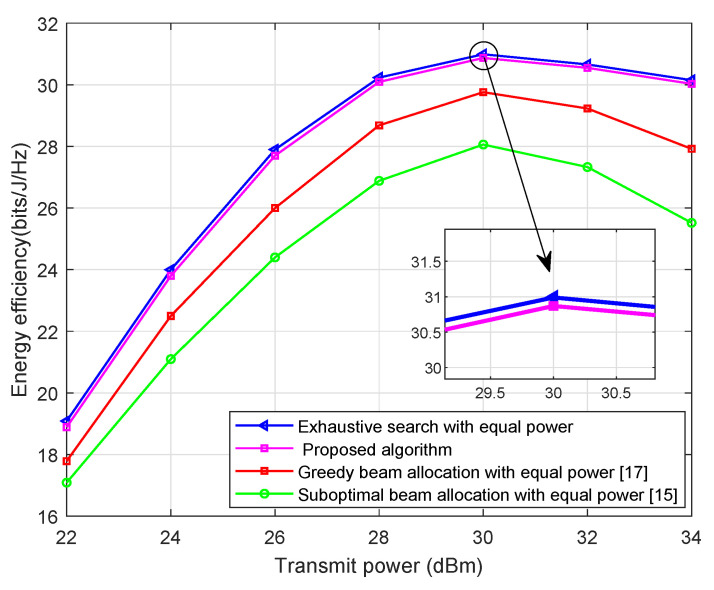
EE versus transmit power with α=2, *U* = 20.

Figure 2 shows that the behavior of the proposed algorithm is almost the same as that of ES algorithm under wide transmit powers. For example, about 30.98- and 30.86-bits/J/Hz EE values were obtained by ES and proposed algorithms when the transmit power was 30 dBm. Besides, the result show that our proposed algorithm has better performance than that of the greedy beam allocation method (greedy beam allocation algorithm: First, each user is associated with its candidate beams with the highest directivity. Second, the greedy method is associate with every user *u* to the beam *m* that provides the highest received signal power at each step (i.e., $m_{u}\;=\;\underset{m\in M}{\arg\max}D_{m}\left(\theta_{u}\right)$ )) [17] and suboptimal beam allocation method (suboptimal beam allocation algorithm: It employs a two-step scheme to solve the beam allocation problems. In the first step, each user is associated with the beam with the highest directivity. In the second step, there are *U* users in total, if a beam is associated with more than one user, then it is allocated to the user with the highest received signal power, where U − 1 comparisons are needed to find the highest received signal power) [15]. For example, when transmit power is 30 dBm, the system EE by the proposed algorithm is higher than the greedy beam allocation method and suboptimal beam allocation method approximately by 1.23- and 2.91-bits/J/Hz, respectively. As expected, the system EE achieved by the four algorithms is slightly decreased when the transmit power goes large. This phenomenon is attributed to the transmit power increase which causes the the total power consumption increase, and leads to the decrease of the system’s EE.

**Figure 3:**
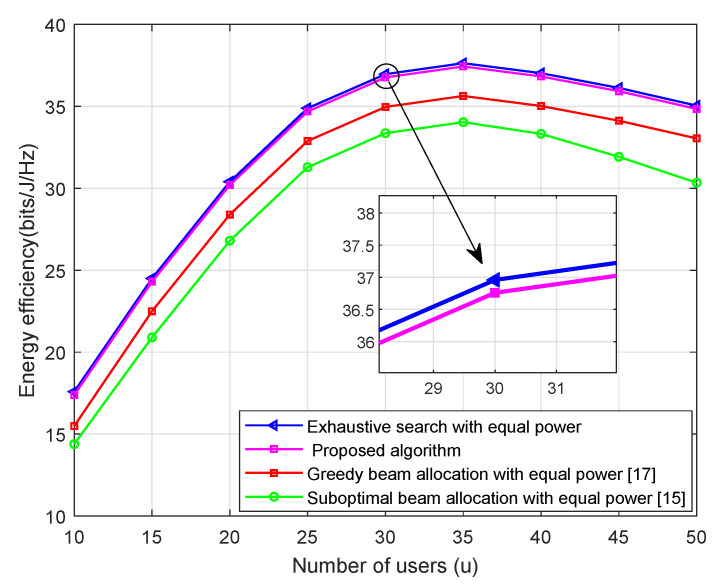
EE versus the number of users with α=2, $P_{t}$ = 30 dBm.

Figure 3 show that the system EE of the four algorithms with adifferent active number of users when the transmit power $P_{T}$ = 30 dBm. As can be seen from Figure 3, the proposed algorithm performs almost identically to the ES method, while the greedy beam allocation and suboptimal beam allocation methods are far from the ES. For example, when the active UEs number equal to 30 (U=30), the achieved values of the system’s EE by using the ES method and our proposed algorithm are 36.95- and 36.75-bit/J/Hz, respectively. A total of 98.8% of the optimal system EE is obtained by our proposed algorithm. In addition, the result shows that the proposed algorithm has a better performance compared with the greedy beam allocation method and suboptimal beam allocation scheme. When the active UEs number equal to 30 (U=30), the proposed algorithm increased the system EE of approximately 1.82 bit/J/Hz and 3.42 bit/J/Hz compared with the greedy beam allocation method and suboptimal beam allocation scheme, respectively. As expected, the system EEs from the four algorithms increases when the number of users is small. When the number of users goes large, the system EE has slightly decrease due to the tradeoff between the ergodic sum-rate and power consumption.

**Figure 4:**
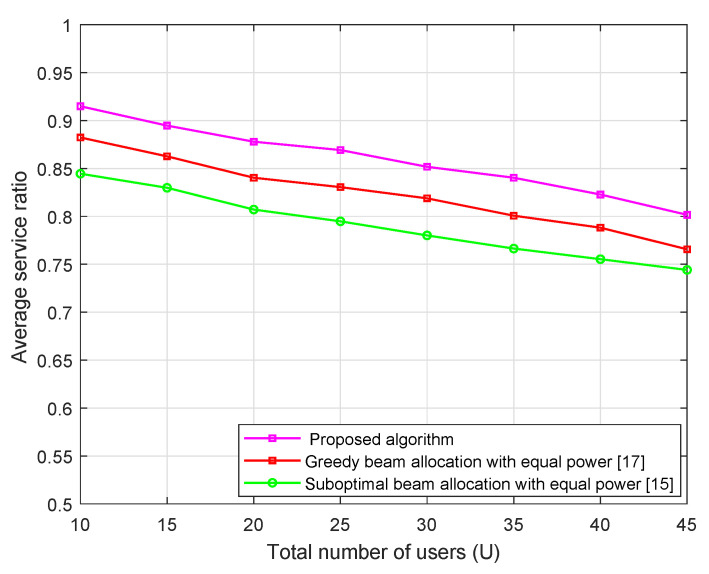
Average service ratio versus total number of users *U* with α=2, *M* = 64, $P_{t}$ = 30 dBm.

Figure 4 shows the average service ratio of three algorithms with the different number of users at transmit power $P_{T}$ = 30 dBm. The figure result show that the average service ratio of proposed algorithm and both the greedy and suboptimal beam allocation algorithms slightly decreases with the increase of active user numbers. This is because the probability that one beam is the best beam for multiple users increases with the number of users *U*. Therefore, the average service ratio slightly decreases as *U* increases. In addition, it can be found that the proposed algorithm shows a better service ratio performance than the greedy beam allocation method and the suboptimal beam allocation scheme.

**Figure 5:**
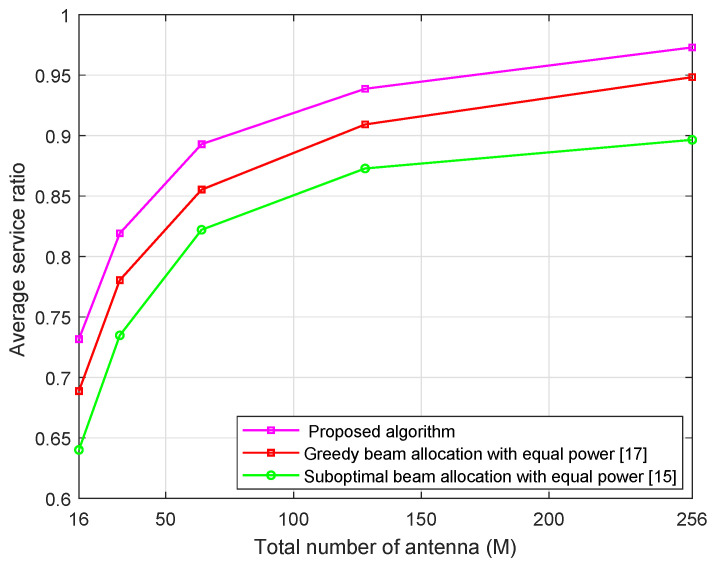
Average service ratio versus total number of antennas *M* with α=2, *U* = 10, $P_{t}$ = 30 dBm.

Figure 5 shows the system average service ratio of three algorithms with the different number of BS antennas at the transmit power $P_{T}$ = 30 dBm. The figure result show that the system average service ratio of the proposed algorithm and both the greedy and suboptimal beam allocation algorithms increase when the number of BS antennas *M* increases. In fact, with a large number of BS antennas *M*, a large number of beams can be formed and thus the number of served users is enlarged. As a result, the average service ratio increase as number of BS antennas *M* increases. In addition, we can find that the proposed algorithm shows a higher performance in terms of average service ratio than the greedy beam allocation algorithm with equal power and the suboptimal beam allocation algorithm with equal power.

**Figure 6:**
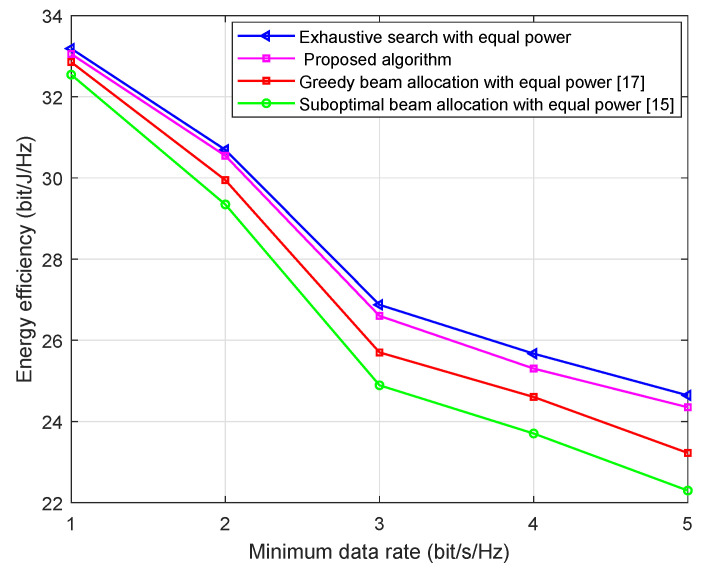
EE versus the required minimum data rate $R_{m,u}^{\min}$ with α=2, $P_{t}$ = 30 dBm.

Figure 6 shows the system EE versus the required minimum data rate $R_{m,u}^{\min}$. The figure result show that the system EE of the compared four algorithms decrease as the minimum data rate $R_{m,u}^{\min}$ increases. When the minimum data rate $R_{m,u}^{\min}$ increases, the QoS constraints is maintained by the required multiple transmit antennas and larger power. This introduces a substantial decrease to the system EE. The gap between the four beam allocation schemes enlarges significantly as the minimum data rate $R_{m,u}^{\min}$ increases. Again, the proposed algorithm provided better system EE than the greedy beam allocation method and suboptimal beam allocation scheme in terms of the system’s EE performance at the minimum data rate $R_{m,u}^{\min}=5$ bit/s/Hz.

### 4.2. Computational Complexity Analysis

In this part, we analyze the computational complexity of our proposed algorithm. For the best beam selection, we used O(M) operations to compute formula (18). Then, we used O(MU), O(U), and O(M) operations to update Lagrange multipliers in (19). Therefore, the number of worst operations for the best beam to select and associated user pairing is O(MU) in each iteration. Similarly, we used O(MU) and O(U) operations to update Lagrange multipliers in (30), and used O(MU) operations to compute formula (29). Hence, the total number of operations for obtaining optimal power is $O(MUT_{\max})$. Therefore, the total complexity of our proposed algorithm is $O(M^{2}U^{2}T_{\max})$. The complexity of the ES method is $T_{\max}O\left(C_{M}^{U}\right)$, where $T_{\max}$ is the total number of iterations and $O\left(C_{M}^{U}\right)\;>>\;O\left(M^{2}U^{2}\right)$. Our proposed algorithm have a very low computational complexity compared to the ES method.

## 5. Conclusions

In this paper, we studied the problem of beam allocation and power optimization in downlink multiuser massive MIMO system working at a mmWave frequency band. The aim is to reduce the hardware cost and power consumption while the system’s EE gains improvement without obvious system performance loss. We decomposed the original non-convex optimization problem into two sub-problems, which were solved using convex optimization techniques. The two sub-problems were beam allocation and power optimization problems. For the beam allocation, we proposed an algorithm based on convex optimization technique. For the power optimization, first the non-convex problem converted into a convex problem by using a quadratic transformation scheme. Then, we used Lagrange dual and sub-gradient methods to solve the optimization problem. In our experiments, the proposed algorithm produced promising results and achieved comparable performance compared to the ES algorithm at a very low computational complexity. Furthermore, the proposed algorithm had a better performance than a greedy beam allocation method and suboptimal beam allocation scheme.

## Figures and Tables

**Table 1:** Simulation parameters setting. UE: User; BS: Base station; RF: Radio frequency.

Parameters	Values
No. of UEs in cell (U)	10∼50
Maximum number of BS antennas (M)	256
Cell radius	150 m
mmWave frequency $\left(f_{c}\right)$	28 GHz
Maximum transmission power $\left(P_{t}\right)$	43 dBm
convergence accuracy (ε)	0.001
$R_{m,u}^{\min}$	2 bits/s/Hz
maximum number of iterations $\left(T_{\max}\right)$	30
power consumption of RF circuit (γ)	250 mW
Noise spectral density $\left(\sigma_{0}^{2}\right)$	−174 dBm/Hz

## Data Availability

No data available.

## Sample Availability

Samples of the compounds are available from the authors.

## Abbreviations

The following abbreviations are used in this manuscript:

5G	Fifth-Generation
MIMO	Multiple-Input Multiple-Output
ES	Exhaustive Search
mmWave	Millimeter Wave
RF	Radio-Frequency
BS	Base Staion
EE	Energy-Efficiency
LOS	Line of Sight
UE	User
